# Evaluating the versatility of EEG models generated from motor imagery tasks: An exploratory investigation on upper-limb elbow-centered motor imagery tasks

**DOI:** 10.1371/journal.pone.0188293

**Published:** 2017-11-29

**Authors:** Xin Zhang, Xinyi Yong, Carlo Menon

**Affiliations:** Menrva Research Group, Schools of Mechatronic Systems Engineering and Engineering Science, Simon Fraser University, Metro Vancouver, British Columbia, Canada; University of Minnesota, UNITED STATES

## Abstract

Electroencephalography (EEG) has recently been considered for use in rehabilitation of people with motor deficits. EEG data from the motor imagery of different body movements have been used, for instance, as an EEG-based control method to send commands to rehabilitation devices that assist people to perform a variety of different motor tasks. However, it is both time and effort consuming to go through data collection and model training for every rehabilitation task. In this paper, we investigate the possibility of using an EEG model from one type of motor imagery (e.g.: elbow extension and flexion) to classify EEG from other types of motor imagery activities (e.g.: open a drawer). In order to study the problem, we focused on the elbow joint. Specifically, nine kinesthetic motor imagery tasks involving the elbow were investigated in twelve healthy individuals who participated in the study. While results reported that models from goal-oriented motor imagery tasks had higher accuracy than models from the simple joint tasks in intra-task testing (e.g., model from elbow extension and flexion task was tested on EEG data collected from elbow extension and flexion task), models from simple joint tasks had higher accuracies than the others in inter-task testing (e.g., model from elbow extension and flexion task tested on EEG data collected from drawer opening task). Simple single joint motor imagery tasks could, therefore, be considered for training models to potentially reduce the number of repetitive data acquisitions and model training in rehabilitation applications.

## Introduction

Several BCIs are based on electroencephalography (EEG). EEG measures the electric brain activity caused by the flow of electric currents during the synaptic excitations of the dendrites in the neurons. [1]. Recently, research on EEG controlled system has become particularly active, as EEG measurement is non-invasive and easy to set up [2–6].

Different EEG-based control approaches have been explored in different populations to assist individuals to reacquire the basic abilities for communication [7] and mobility (e.g., control of neuroprostheses [8–10] and wheelchairs [11]). Recently, research groups have also explored the use of EEG controlled systems in stroke rehabilitation, in order to encourage users to be actively engaged during the rehabilitation process [3][12]. A current challenge is to develop EEG controlled systems for a large number of tasks with high accuracy [4]. To overcome this problem, the building of binary classification models for each task has been investigated [13]. However, repetitively acquiring EEG data and building EEG models for each task does require considerable effort on the part of the user and is also time-consuming. A possible solution is to build a general EEG model based on EEG data of a specific movement, which can be reused in different but similar training tasks (general model approach, GM for short).

Motor imagery is a common method for EEG controls in the literature [4][14]. Motor imaginary can be either goal-oriented or be related to a single joint. Goal-oriented motor imagery refers to imagery on context-specific movements, such as grasping a glass of water for drinking or eating with a spoon [15]. On the other hand, single joint motor imagery, as referred to in this paper, consists of imagining a single joint movement that is not goal-oriented or has a specific meaningful purpose. Examples of single joint motor imagery include imagining flexing or extending the elbow, the wrist, or another joint without grasping an object or any specific function [15].

Studies have shown that practice of goal-oriented tasks after stroke produces long-lasting cortical reorganization compared to traditional stroke rehabilitation[15][16][17]. Additionally, Boyd et al. demonstrated that goal-oriented task training with the hemiparetic arm resulted in both functional reorganization of both motor cortices and a larger motor learning-related change after stroke[18].

Despite the importance of goal-oriented tasks in stroke rehabilitation, most existing EEG controlled systems were developed to perform simple movements rather than goal-oriented tasks (see Table 1). Only a few studies considered goal-oriented tasks (e.g. Frisoli, A. et al. [19], Royer, AS. et al.[20], Min, BK. et.al[21]).

**Table 1:** Examples of different EEG control setup and tasks used in the literature.

Bibliography	Feedback	EEG Classes
**Wolpaw et al. 2004** [22]	EEG+Visual	8-Class: By combining Vertical and Horizontal control to select 8 targets
**Meng et al. 2008** [23]	EEG + Visual + FES	2-Class: MI (Wrist/Hand) vs Rest
**Buch et al. 2008**[24]	EEG + Visual + Orthosis	2-Class: MI (Grasp) vs MI (Open)
**Daly et al. 2009**[25]	EEG + Visual + FES	2-Class: MI/AT (Finger Extension) vs Relax
**Ying Gu et al. 2009**[26]	EEG	4-Class: MI of finger/wrist with different moving speed
**Prasad et al. 2010**[27]	EEG + Visual	2-Class: MI Left vs MI Right (Arm/Hand)
**Tan et al. 2010** [28]	EEG + Visual + NES	2-Class: MI (Hand) vs Rest
**Ang et al. 2010**[29]	EEG + Visual + Robot	2-Class: MI/AT (Grasp) vs Rest
**Broetz et al. 2010**[30]	EEG + Visual + Orthosis	2-Class: MI/AT (Grasp) vs MI/AT (Open)
**Tam et al. 2011** [31]	EEG + Visual + FES	2-Class: MI (Wrist) vs Rest
**Gomez-Rodriguez et al. 2010**[32]	EEG + Visual + Robot	2-Class: MI (Elbow Flexion/Extension) vs Rest
**Shindo et al. 2011**[33]	EEG + Visual + Orthosis	2-Class: MI (Open Hand) vs Rest
**Ortner et al. 2012**[34]	EEG + Visual	2-Class: MI Left vs MI Right (Hand)
**Kaiser et al. 2012**[35]	EEG + Visual	2-Class: MI/AT (Grasp) vs Rest
**Cincotti et al. 2012**[36]	EEG + EMG + FES	2-Class: MI/AT (Grasp/Finger Extension) vs Relax
**Frisoli et al. 2012**[19]	EEG + Arm Exoskeleton + Kinect + Eye-Tracker	2-Class: MI (Right Arm) vs Rest
**Vuckovic et al. 2012**[37]	EEG	4-Class: MI on both wrist movement
**Ramos-Murguialday et al. 2013**[38]	EEG + Orthosis	2-Class: AT (Reach & Grasp) vs Rest
**Young et al. 2014**[39]	EEG + Visual + FES + TS	2-Class: AT (Open + Close Hand) vs Rest
**Ang et al. 2015**[40]	EEG + Visual + Robot	2-Class: MI (Grasp) vs Rest
**Pinto et al.2015**[41]	EEG	2-Class: Action vs Rest; 4-Class: L-R motor, L-R MI
**Ibáñez et al. 2015**[42]	EEG+FES	2-Class: AT(Elbow) vs Rest
**Yong et al. 2015**[13]	EEG Offline Analysis	4-Class: MI(Grasp, Elbow, Reach&Grasp) vs Rest
**Elnady et al. 2015**[43]	EEG + Exoskeleton + FES	2-Class: MI (Grasp) vs Rest
**Edelman et al. 2016**[44]	EEG	4-Class: MI on one hand movement

MI: motor imagery; AT: attempted movement; NES: neuromuscular electrical stimulation; TS: tongue stimulation; S: stroke volunteers; H: healthy individuals; sess: session(s)

Recent literature has shown that the motor imagery (MI) of goal-oriented movements is better than non-goal-oriented movements in terms of achieving higher EEG control accuracy [13]. However, in practical rehabilitation applications, participants would have to spend time and effort in repetitive data acquisition and model training for each different goal-oriented task. On the other hand, the use of a GM could potentially drastically reduce the training time as the training would be done on a single task. However, it is not known whether an EEG model trained using the EEG signals of the motor imagery of a single upper extremity movement (e.g., elbow flexion and extension) could be used to classify the motor imagery of similar other movements (e.g., opening a door, combing hair, placing a ball into a basket, etc.). To the best of the authors’ knowledge, it is also not known which movement would work best to generate the GM. The investigation into a model can be reused in different training tasks is an important problem to be addressed especially in EEG controlled rehabilitation applications, where each goal-oriented movement is generally functionally different from the others.

The main goal of this exploratory study is to determine which motor imagery task is the most suitable to make the EEG model versatile during EEG acquisition, i.e. have the highest inter-task test accuracy. Specifically, the versatility of nine different motor imagery tasks was considered in this paper. In this context, versatility means that the EEG model generated from one specific motor imagery task leads to good performance when tested on the EEG data of other motor imagery tasks. In this study, six classification methods were used to generate the EEG models of the nine predefined motor imagery tasks. Then, the EEG data from other eight motor imagery tasks were used to test the inter-task test accuracy of the EEG model. Finally, a statistical analysis was performed to determine which motor imagery task was the most versatile when used as a GM.

Given the complexity of the problem, this exploratory study focuses only on upper-extremity movements to simplify the investigation. Specifically, all the tasks were selected to be centered on the elbow joint.

## Methods

All the methods within this study were in compliance with the Declaration of Helsinki. The study was also approved by the Simon Fraser University (SFU) Office of Research Ethics.

In this study, 12 participants (aged 20–33 years old, 10 males and 2 females) agreed to join the study. All the participants signed informed consent forms before taking part in the experiment. Each individual was seated in front of a computer monitor, which provided a simple Graphical User Interface (GUI) that displayed pictures or cues to the participant.

### Experimental protocol

**Fig 1:**
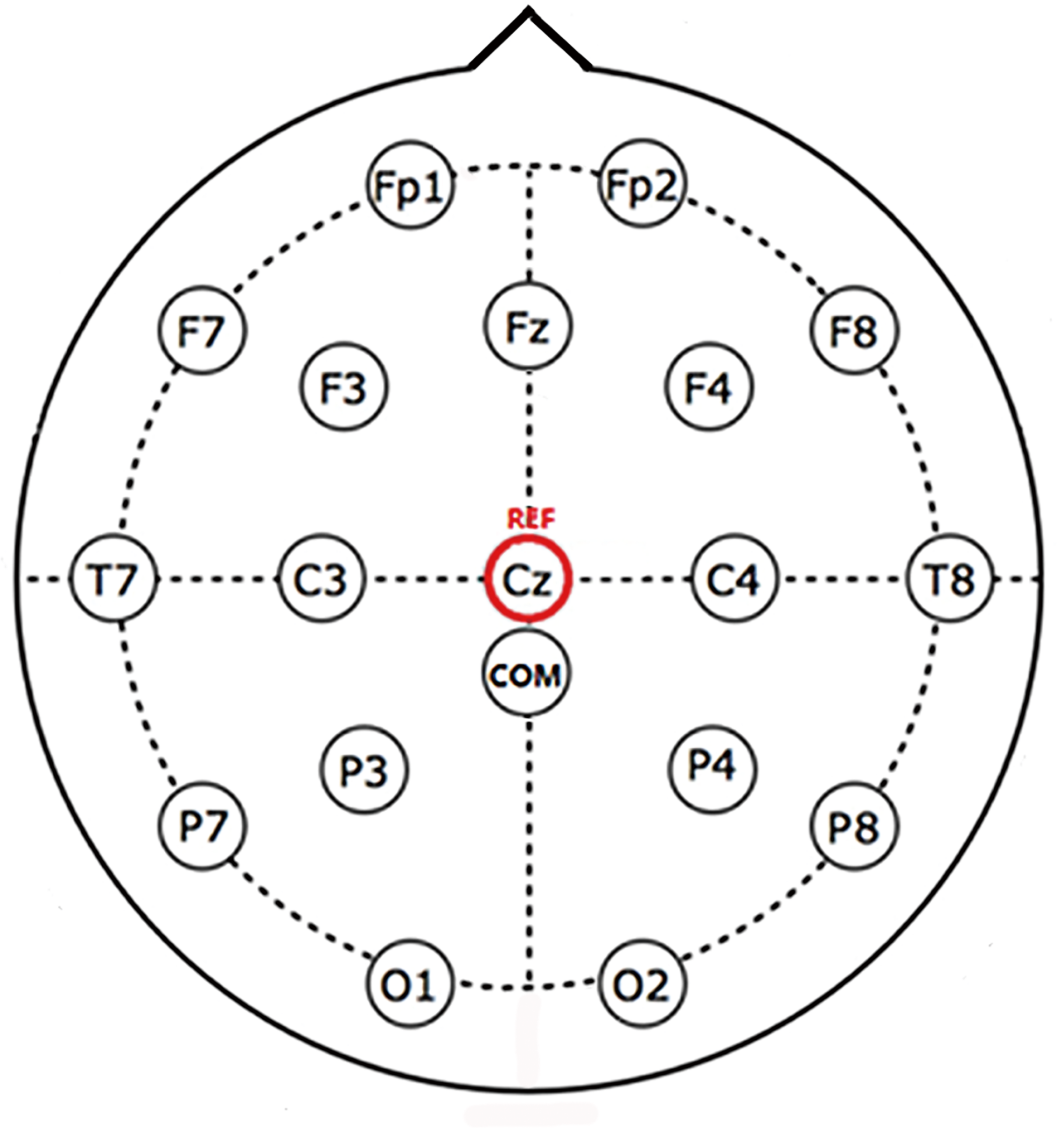
Contact montage of the EEG system in the experiment, 17 channels was used. Cz was defined as the reference contact by the EGI system, COM was the common ground contact.

A 32-channel, EGI Geodesic N400 system (Electrical Geodesics Inc., Eugene, OR, USA) was used to acquire the EEG data from the participants. EEG data were amplified and recorded at a sampling rate of 1 kHz. The electrode contact sites are shown in Fig 1. 17 channels were used in this study, as the remaining channels were located on the face (the EGI cap does not allow to re-position the electrodes). All participants were requested to wear the EGI sensor net for approximately 40 minutes during this experiment. During the experiment, the participants could take a break if desired.

**Fig 2:**
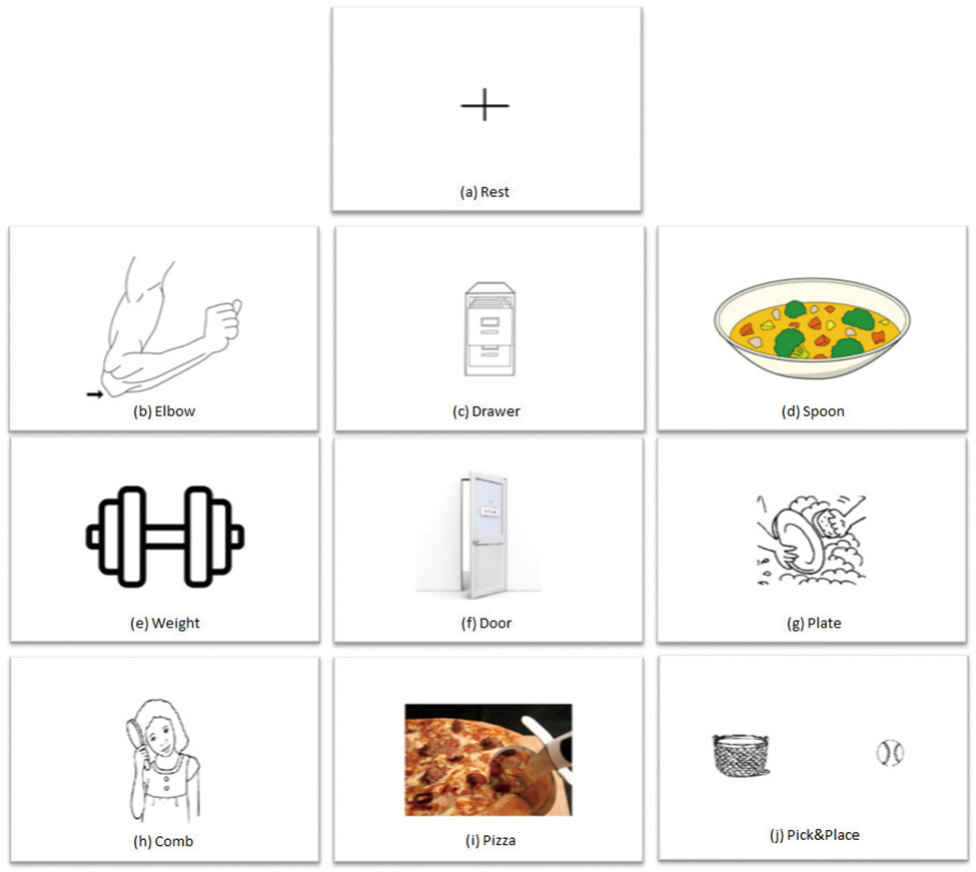
Picture of the tasks that were used in the Stimulus Presentation tasks where: (a)Rest Task, rest and stay alerted; (b)Elbow Task, imagine elbow flexion and extension; (c)Drawer Task, imagine opening and closing a drawer; (d)Soup Task, imagine drinking soup with a spoon; (e)Weight Task, imagine lifting and putting down a dumbbell; (f)Door Task, imagine opening and closing a door; (g)Plate Task, imagine cleaning a plate; (h)Comb Task, imagine combing hair; (i)Pizza Task, imagine cutting a pizza with a pizza cutter; and (j) Pick &Place Task, imagine picking up a ball and put it into a basket.

EEG data were collected using the Stimulus Presentation mode in BCI2000[45]. During Stimulus Presentation, customized pictures were shown on the screen while the EEG signals were recorded and filtered with a bandpass filter of 0.1–40 Hz. In this study, the pictures for ten different tasks were randomly selected and displayed on the screen. These pictures are presented in Fig 2. The participants were asked to repetitively perform the kinaesthetic motor imagery task displayed on the screen for 4 seconds without actually moving. Kinaesthetic motor imagery means that the participants were required to perform imaginary movement by focusing on imagining the sensation of the movement[46].

In this study, nine motor imagery tasks were chosen as upper limb movements. Tasks were selected to primarily involve the elbow joint. These motor imagery tasks can be divided into three main categories: 1) simple joint task that do not have any context meaning. In this paper, we chose Elbow Task, Drawer Task, and Weight Task; 2) simple elbow joint tasks that are commonly executed in daily life and require a relatively low level of synergy of other joints. In this paper we chose Door Task, Plate Task, and Comb Task; and 3) goal-oriented tasks, which require trajectory planning and multiple joint synergies. In this paper, we chose Soup Task, Pizza Task, and Pick&Place Task. The specific instructions given to the participants with respect to the ten tasks are summarized below:

1. Rest (Fig 2(A)): rest while looking at the center of the cross;
2. Elbow task (Fig 2(B)): kinaesthetically imagine flexing and extending the elbow of the dominant arm;
3. Drawer task (Fig 2(C)): kinaesthetically imagine opening and closing a drawer with the dominant hand;
4. Soup task (Fig 2(D)): kinaesthetically imagine getting a spoonful of soup and drinking the soup using the dominant hand;
5. Weight task (Fig 2(E)): kinaesthetically imagine lifting and putting down a dumbbell with the dominant hand;
6. Door task (Fig 2(F)): kinaesthetically imagine opening and closing door with the dominant hand on the door knob;
7. Plate task (Fig 2(G)): kinaesthetically imagine cleaning a plate with only elbow extension and flexion movement;
8. Comb task (Fig 2(H)): kinaesthetically imagine combing hair with the dominant hand.
9. Pizza task (Fig 2(I)): kinaesthetically imagine cutting a pizza with a pizza cutter with the dominant hand;
10. Pick&Place Task (Fig 2(J)): kinaesthetically imagine picking a ball and placing it into a basket with the dominant hand.

During the Stimulus Presentation, each picture was displayed on the screen for 4–6 seconds, followed by 4–6 seconds of rest, and the timing was randomized by the software in order to prevent participants from adapting. When the picture was displayed on the screen, the participant was requested to perform motor imagery of the corresponding task repetitively for 1–2 repetitions. For each participant, the test consisted of 15 consecutive runs. Each run consisted of 4 Rest, 4 Elbow Tasks and 16 other tasks (2 for each of the remaining tasks). Each run lasted for approximately 3 minutes. Each participant was requested to complete 15 runs and he/she could rest for as long as was needed between two runs. The participants were required to follow the stimulus on the screen. While the picture was on the screen, the participants were required to perform the respective tasks repetitively for 2–3 repetitions. As in many MI studies reported in the literature, electromyography (EMG) was not recorded [47][48][49]. To ensure compliance to the protocol, we had one observer monitor the participants to ensure they were not moving during the task. In the case of the slightest movement, the recorded data were disregarded, and the participant was asked to repeat the experiment.

### Participants

Twelve healthy participants, aged between 20 and 33 participated in this study. Their demographic data are presented in Table 2.

**Table 2:** Demographic data for the participants.

Participants	Gender	Age	Dominant Hand
**H01**	M	27	Right
**H02**	F	31	Right
**H03**	M	21	Right
**H04**	M	30	Right
**H05**	M	26	Left
**H06**	M	20	Right
**H07**	M	33	Right
**H08**	M	23	Right
**H09**	F	33	Right
**H10**	M	28	Right
**H11**	M	24	Right
**H12**	M	21	Right

### Feature extraction and classification

The data acquired were analyzed using BCILAB[50], a BCI toolbox based on Matlab. The data were first resampled at 250 Hz. Then, a finite impulse response (FIR) bandpass filter was used to filter out the 6–35 Hz frequency band. By band-pass filtering, the data, ocular artifacts and other undesired frequency components of the EEG data were minimized. This frequency band covers the mu and beta rhythms, which have been reported to desynchronize during motor imagery [51]. According to the literature, the band power changes of the mu and beta rhythms have been used in BCI systems to classify EEG signals related to motor imagery [52–54]. Those activities are localized in the mu (7–13 Hz) and beta bands (13–30 Hz). Therefore, band power (BP) of a certain band frequency can be used as a basic feature for classification [51,55]. However, ERD/ERS signals could be overlapped in time and space by multiple signals from different brain tasks. For this reason, in some cases, it may not be sufficient to use simple methods such as a band pass filter to extract the desired band power. The literature suggests that spatial filters, like common spatial pattern (CSP), could be appropriate [56]. The performance of spatial filters is dependent on its operational frequency band. Therefore, we also included filter bank CSP (FBCSP) to avoid this potential problem [57,58].

As each participant had a different reaction time to the stimulus, nine different epoch periods were extracted from the EEG data to find out the optimal epoch that led to the best EEG control performance. The different epochs used are presented in Table 3.

**Table 3:** Epoch periods used in data analysis.

Epoch ID	Epoch Period*
**1**	0.5–2.5s
**2**	1-3s
**3**	1.5–3.5s
**4**	2-4s
**5**	2.5–4.5s
**6**	3-5s
**7**	1-3s
**8**	1-4s
**9**	1-5s

*Refer to the time after the stimulus was shown on the screen

In this paper, BP[59], CSP[53] and FBCSP [57] were used as feature extraction algorithms to extract features, for each EEG epoch. Detailed information is presented in Table 4.

**Table 4:** Feature setting for model training.

Algorithm	Frequency Band	Feature Dimension
BP	6-32Hz	17
CSP	6-32Hz	6
FBCSP	6-15Hz; 15-25Hz; 25-32Hz	18

The features were then sent to classifiers. Since we wanted to evaluate the influence of different motor imageries in this paper, classifiers were limited with basic classifiers. In this study, linear discriminant analysis (LDA) and dual-augmented lagrangian (DAL) method were used for classification. All the classifiers were regularized during training. For LDA, analytical covariance shrinkage was used for regularization [60]. For DAL, dual-spectral logistic norm was used for regularization, with grid searching λ from 2^−15^ to 2^10^, the step size was 2 times [61]. A binary classifier was generated for the EEG features obtained from Rest Task data and one of the Tasks (b)-(j) respectively. A 5×5 cross-validation method was used to validate the performance of the classifiers.

We used 3 features (i.e. BP, CSP, and FBCSP) and 2 classifiers (LDA, DAL) which resulted in 6 models per epoch for each participant. We considered 9 epochs, which resulted in 54 different models (3×2×9 = 54). We selected the best model for each motor imagery task for each participant. Each participant performed 9 different tasks, and we invited 12 participants. We, therefore, obtained 108 models in total (9×12 = 108). By doing this, we set a uniform objective classification standard for all nine different motor imagery tasks. The performance of the models from these motor imagery tasks is presented in the following sections.

### Model training and testing

The main goal of the work was to assess the versatility of the EEG models derived from different motor imagery tasks. We studied this in the inter-task problem, where the model generated from one type of motor imagery task was tested with data from another motor imagery task. The data were collected to investigate this inter-task problem. Specifically, 30 trials (T) for each of the 9 motor imagery tasks (i.e. T_1_ -T_9_) were collected. For each task, the data were randomized. Furthermore, 60 trials of rest were recorded. After randomization, they were divided in two groups: training (R_TR_) and testing (R_TE_). Therefore, a total number of 330 trials (i.e. 30 trials × 9 motor imagery tasks + 30 rest for training (R_TR_) + 30 rest for testing (R_TE_)) were recorded.

During training, 9 two-class models were created for each participant. Each model, corresponding to a single task, was trained using the 30 trials of rest (R_TR_) collected for training purposes (class 1) + the 30 trials related to the single task in question (class 2). Specifically, Model 1 (*m*_1_INTER_) was trained using T_1_ and R_TR_, model 2 (*m*_2_INTER_) was trained using T_2_ and R_TR_, etc. Table 5 shows the training datasets for each model. A 5-fold cross-validation was used to generate the models during training.

**Table 5:** Data usage in training models for inter-task problem.

Model Name	*m*_1_INTER_	*m*_2_INTER_	*m*_3_INTER_	*m*_4_INTER_	*m*_5_INTER_	*m*_6_INTER_	*m*_7_INTER_	*m*_8_INTER_	*m*_9_INTER_
**Data Used**	T_1_ and R_TR_	T_2_ and R_TR_	T_3_ and R_TR_	T_4_ and R_TR_	T_5_ and R_TR_	T_6_ and R_TR_	T_7_ and R_TR_	T_8_ and R_TR_	T_1_ and R_TR_

For testing, each model was tested with data collected for the other models. Specifically, m_1_ was tested with 8 testing datasets, the first being T_2_+R_TE_, the second being, T_3_+R_TE_, the third T_4_+R_TE_, etc. Table 6 shows the data usage in testing datasets.

**Table 6:** Data usage in the inter-task testing.

Model Name	Elbow	Drawer	Spoon	Weight	Door	Plate	Comb	Pizza	Pick& Place
***m*_1_INTER_**	----	T_2_+R_TE_	T_3_+R_TE_	T_4_+R_TE_	T_5_+R_TE_	T_6_+R_TE_	T_7_+R_TE_	T_8_+R_TE_	T_9_+R_TE_
***m*_2_INTER_**	T_1_+R_TE_	---	T_3_+R_TE_	T_4_+R_TE_	T_5_+R_TE_	T_6_+R_TE_	T_7_+R_TE_	T_8_+R_TE_	T_9_+R_TE_
***m*_3_INTER_**	T_1_+R_TE_	T_2_+R_TE_	---	T_4_+R_TE_	T_5_+R_TE_	T_6_+R_TE_	T_7_+R_TE_	T_8_+R_TE_	T_9_+R_TE_
***m*_4_INTER_**	T_1_+R_TE_	T_2_+R_TE_	T_3_+R_TE_	---	T_5_+R_TE_	T_6_+R_TE_	T_7_+R_TE_	T_8_+R_TE_	T_9_+R_TE_
***m*_5_INTER_**	T_1_+R_TE_	T_2_+R_TE_	T_3_+R_TE_	T_4_+R_TE_	---	T_6_+R_TE_	T_7_+R_TE_	T_8_+R_TE_	T_9_+R_TE_
***m*_6_INTER_**	T_1_+R_TE_	T_2_+R_TE_	T_3_+R_TE_	T_4_+R_TE_	T_5_+R_TE_	---	T_7_+R_TE_	T_8_+R_TE_	T_9_+R_TE_
***m*_7_INTER_**	T_1_+R_TE_	T_2_+R_TE_	T_3_+R_TE_	T_4_+R_TE_	T_5_+R_TE_	T_6_+R_TE_	---	T_8_+R_TE_	T_9_+R_TE_
***m*_8_INTER_**	T_1_+R_TE_	T_2_+R_TE_	T_3_+R_TE_	T_4_+R_TE_	T_5_+R_TE_	T_6_+R_TE_	T_7_+R_TE_	---	T_9_+R_TE_
***m*_9_INTER_**	T_1_+R_TE_	T_2_+R_TE_	T_3_+R_TE_	T_4_+R_TE_	T_5_+R_TE_	T_6_+R_TE_	T_7_+R_TE_	T_8_+R_TE_	---

Before running the inter-task problem, the authors wanted to ensure that the considered BP/CSP/FBCSP+LDA/DAL method was a suitable method for the motor imagery tasks considered. Therefore, an intra-task problem was first addressed. In this case, each task had to be tested with data collected from the same motor imagery task (e.g. a model trained with T_1_ could not be tested with T_2_ as for the inter-task case as T_1_ and T_1_ were datasets related to different tasks, thus not suitable for the intra-task case). For this reason, each of the 30 trials was divided in training and testing datasets for the intra-task case. Specifically, 24 trials of each motor imagery task (e.g. T_1_TR_) together with 24 trials of Rest Task (R_intra_TR_) were used for training. The remaining six trials of the same motor imagery task (e.g. T_1_TE_) together with 6 trials of Rest Task (R_intra_TE_) were used for testing. Table 7 shows the training and testing dataset for each model.

**Table 7:** Training and testing datasets for the intra-task problem.

Model Name	Data used in training	Data used in Testing
***m*_1_INTRA_**	T_1_TR_ and R_intra_TR_	T_1_TE_ and R_intra_TE_
***m*_1_INTRA_**	T_2_TR_ and R_intra_TR_	T_2_TE_ and R_intra_TE_
***m*_1_INTRA_**	T_3_TR_ and R_intra_TR_	T_3_TE_ and R_intra_TE_
***m*_1_INTRA_**	T_4_TR_ and R_intra_TR_	T_4_TE_ and R_intra_TE_
***m*_1_INTRA_**	T_5_TR_ and R_intra_TR_	T_5_TE_ and R_intra_TE_
***m*_1_INTRA_**	T_6_TR_ and R_intra_TR_	T_6_TE_ and R_intra_TE_
***m*_1_INTRA_**	T_7_TR_ and R_intra_TR_	T_7_TE_ and R_intra_TE_
***m*_1_INTRA_**	T_8_TR_ and R_intra_TR_	T_8_TE_ and R_intra_TE_
***m*_1_INTRA_**	T_9_TR_ and R_intra_TR_	T_9_TE_ and R_intra_TE_

### The coefficient of determination (R2 value)

The coefficient of determination (R^2^ value) is a statistical measure computed over a pair of sample distributions, which measures how strongly the means of the two distributions differ in relation to variance [62]. In a BCI context, the R^2^ value is computed over signals that have been measured under two different task conditions. It represents the fraction of the total signal variance caused by different tasks [62]. It is a measure of how well the task condition is reflected in the brain activities [62].

The R^2^ value at each electrode location was computed for all participants and all combinations of different tasks in order to investigate the topographical distribution on the scalp of the difference between rest and the other imaginary tasks. The frequency that generated the highest R^2^ value was used to generate the topography. The 6-32Hz frequency component was considered for this representation as motor imagery was investigated.

## Results

This section reports the results of the intra-task problem to assess the validity of the BP/CSP/FBCSP+LDA/DAL method before addressing the inter-task problem which is the main focus of this work.

### Inter-task problem: Cross-validation results using the training dataset

**Fig 3:**
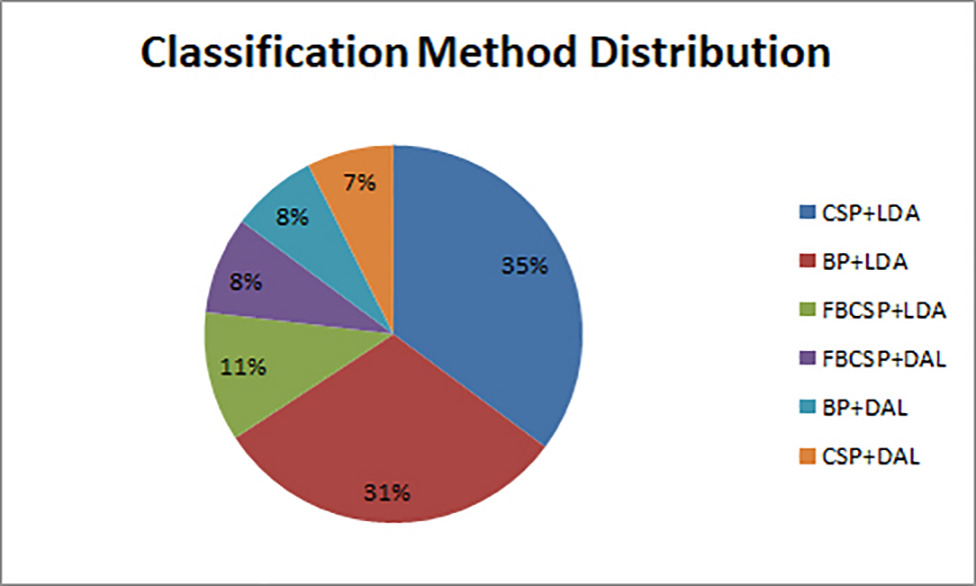
Distribution of the classification method of the highest cross-validation accuracy.

For the inter-task problem the models were generated according to Table 5. Fig 3 summarizes the distribution of the feature algorithms and classifiers used to obtain the model. Among all the features and classifiers, CSP together with LDA was the most common combination: it took 35% of all the 108 models. BP feature with LDA contributed 30% to all the models.

The cross-validation accuracy achieved for each of the nine EEG models and participants is shown in Table 8. This table reports the cross-validation accuracy with the highest value obtained from the optimal combination of the epoch period, feature extraction method and the classifier discussed earlier.

**Table 8:** 5x5 cross-validation accuracy for each participant.

ID	Elbow	Drawer	Spoon	Weight	Door	Plate	Comb	Pizza	Pick &Place	Mean &P
**H1**	0.840	0.757	0.845	0.840	0.817	0.893	0.832	0.893	0.943	0.851&Place
**H2**	0.705	0.748	0.752	0.747	0.758	0.723	0.712	0.775	0.740	0.740&Place
**H3**	0.783	0.803	0.755	0.788	0.823	0.793	0.772	0.822	0.830	0.797&Place
**H4**	0.797	0.743	0.840	0.798	0.802	0.812	0.832	0.905	0.788	0.813&Place
**H5**	0.835	0.817	0.883	0.855	0.878	0.820	0.853	0.903	0.825	0.852&Place
**H6**	0.670	0.732	0.772	0.708	0.717	0.768	0.792	0.738	0.753	0.739&Place
**H7**	0.852	0.848	0.805	0.798	0.850	0.942	0.822	0.907	0.883	0.856&Place
**H8**	0.810	0.800	0.890	0.830	0.860	0.883	0.765	0.878	0.837	0.840&Place
**H9**	0.777	0.787	0.788	0.847	0.792	0.782	0.823	0.787	0.885	0.807&Place
**H10**	0.943	0.952	0.900	0.930	0.928	0.882	0.957	0.930	0.997	0.935&Place
**H11**	0.775	0.733	0.728	0.780	0.695	0.755	0.733	0.712	0.870	0.754&Place
**H12**	0.842	0.802	0.815	0.855	0.738	0.733	0.820	0.912	0.790	0.812&Place

**Fig 4:**
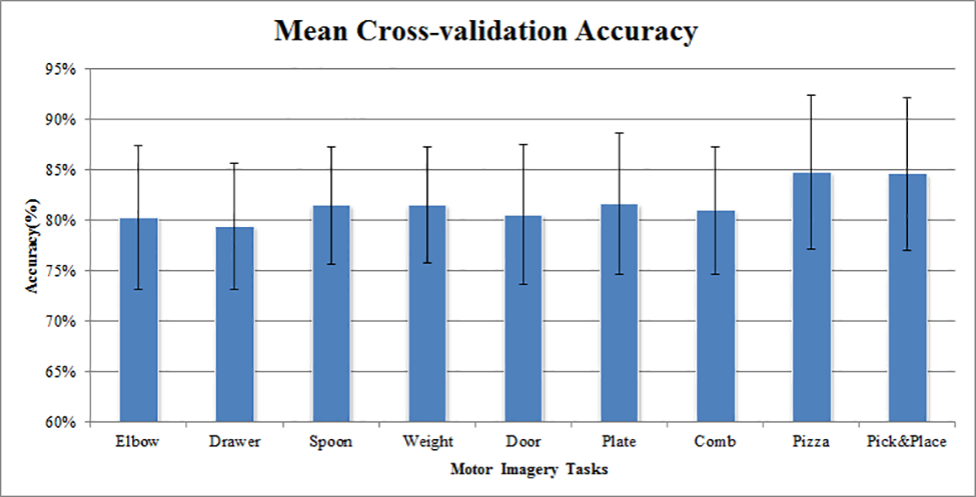
Mean 5×5 cross-validation accuracy for different motor imagery tasks.

As shown in Table 8, the task with the highest cross-validation accuracy was subject-specific. H10 achieved the highest mean cross-validation accuracy (0.935±0.033) among the participants. This participant achieved the highest cross-validation accuracy for the Pick&Place Task (0.997± 0.023). H6, on the other hand, had the lowest cross-validation accuracy (0.739±0.037). The motor imagery task with the highest average cross-validation accuracy is Comb task (0.792± 0.160). Fig 4 shows the 5×5 cross-validation accuracy averaged across participants. The cross-validation accuracy ranges from 0.793±0.062 to 0.847±0.076, with the Pizza Task having the highest cross-validation accuracy and the Drawer Task having the lowest mean cross-validation accuracy. One-way analysis of variance (ANOVA) was used to check the cross-validation accuracy difference among different tasks, no statistical difference was found (p = 0.536).

### Inter-task problem: Testing result

The models were generated and tested as described in Table 6 for testing the results of the inter-task problem. The test accuracy obtained from the inter-task test is summarized in Table 9. More specifically, the model for each motor imagery task was tested on 30 trials of eight other motor imagery tasks. For example, the model generated from Elbow Task was tested with EEG data from all the other tasks, but not from Elbow Task. All test accuracies for all EEG models were greater than 0.5. Table 9 also shows that Weight Task model has the highest average inter-task test accuracy. More specifically, it has the highest average accuracy when tested on data from other motor imagery tasks.

**Table 9:** Inter-task test accuracy summary.

		Test Data (30 trials together with 30trials of Rest Task data)									
		Elbow	Drawer	Spoon	Weight	Door	Plate	Comb	Pizza	Pick& Place	Mean±SD
Model Name	Elbow	---	0.561	0.583	0.607	0.578	0.597	0.589	0.635	0.603	0.594±0.022
	Drawer	0.637	---	0.572	0.571	0.583	0.607	0.578	0.588	0.581	0.589±0.022
	Spoon	0.592	0.535	---	0.533	0.538	0.547	0.535	0.549	0.535	0.545±0.020
	Weight	0.641	0.604	0.617	---	0.565	0.596	0.588	0.626	0.600	0.605±0.024
	Door	0.601	0.561	0.556	0.528	---	0.563	0.533	0.558	0.532	0.554±0.024
	Plate	0.597	0.543	0.531	0.539	0.538	---	0.524	0.551	0.519	0.543±0.024
	Comb	0.637	0.536	0.565	0.568	0.546	0.557	---	0.588	0.538	0.567±0.033
	Pizza	0.615	0.524	0.569	0.536	0.543	0.536	0.540	---	0.532	0.549±0.030
	Pick& Place	0.645	0.563	0.567	0.572	0.554	0.565	0.553	0.586	---	0.576±0.030
	Mean±SD	0.620±0.022	0.553±0.025	0.570±0.024	0.557±0.027	0.556±0.018	0.571±0.026	0.555±0.026	0.585±0.032	0.555±0.034	---

The mean values reported in the last column of Table 9 summarize the averaged inter-task test accuracy for models generated from the nine motor imagery tasks. This indicates the ability of the models to classify EEG data from other motor imagery tasks. The mean values reported in the last row of Table 9 summarize the averaged inter-task test accuracy for EEG data from the nine motor imagery tasks, which indicates the versatility of EEG data for the nine motor imagery tasks. The mean model test accuracy ranges from 0.543±0.023 to 0.605±0.022. The model generated from the Weight task data has the highest mean inter-task test accuracy, while the model generated from Plate Task data has the lowest mean test accuracy. The mean data test accuracy ranges from 0.553±0.025 to 0.620±0.022. The data from Elbow Task has the highest mean inter-task test accuracy and the data from Drawer Task has the lowest mean inter-task test accuracy.

**Fig 5:**
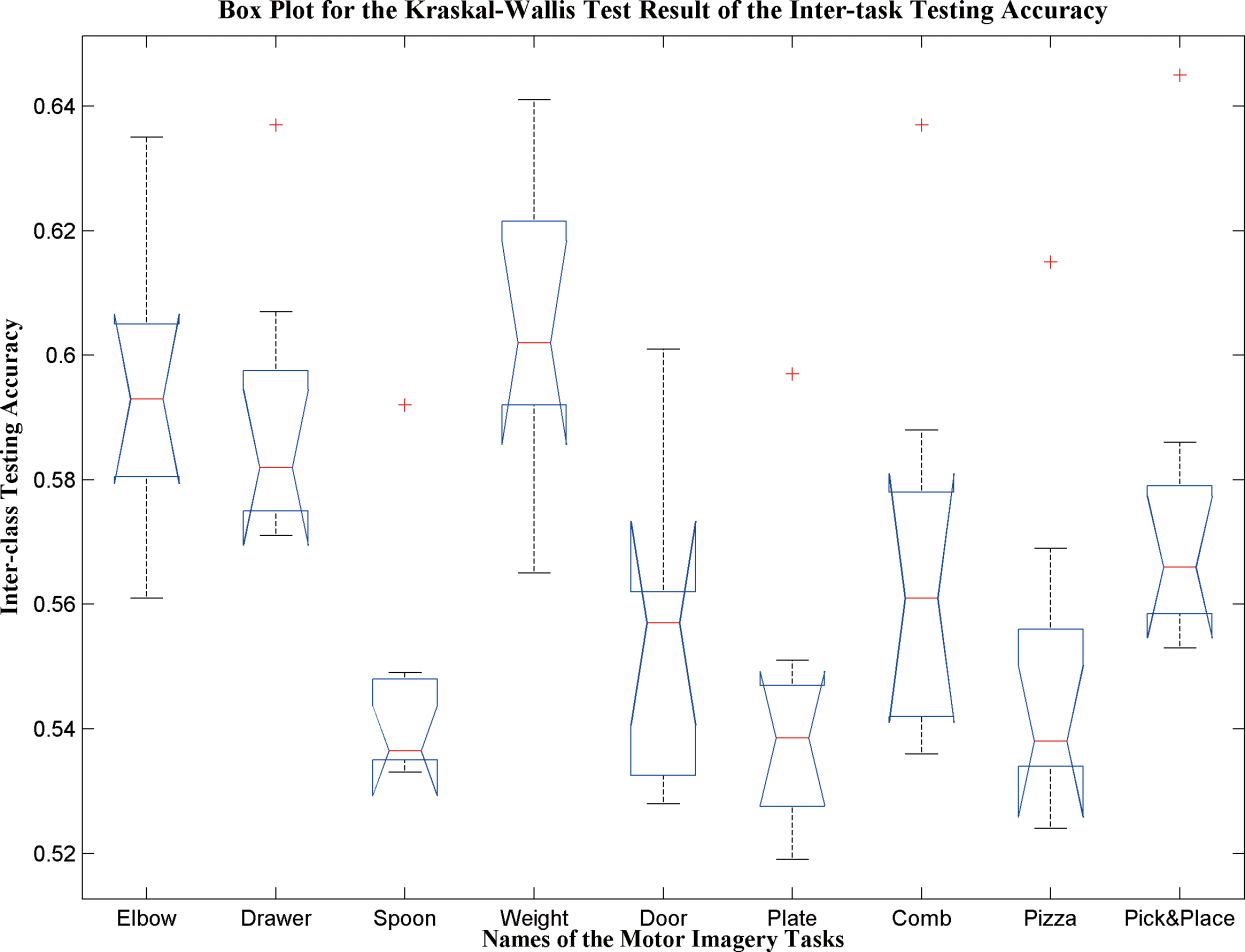
Box plot for the Kruskal-Wallis test result for the inter-task testing accuracy.

A Shapiro-Wilk parametric hypothesis test was performed to test the normality of the test accuracies for different task data in Table 9. The test accuracies for models Drawer, Spoon, Plate, Pizza, Pick&Place are not normally distributed (their *p* values are 0.030, 0.002, 0.030, 0.012, and 0.006 respectively). Kruskal-Wallis test showed the inter-task test accuracy is statistically different (p = 2.6×10^−5^), see Fig 5.

In the post-hoc analysis, Dunn & Sidák’s approach was used [63]. The model from the Weight Task has statistically higher inter-task test accuracy, compared to the model from the Spoon Task, Door Task, Plate Task, and Pizza Task(p<0.05). No statistical difference was found among Elbow Task, Drawer Task, and Weight Task (p>0.05), see Table 10.

**Table 10:** Dunn & Sidák post-hoc analysis of the inter-task testing accuracy. Checkmarks indicate models whose inter-task accuracies are significantly different (p<0.05).

Model Names	Elbow	Drawer	Spoon	Weight	Door	Plate	Comb	Pizza	Pick& Place
Elbow			√			√			
Drawer									
Spoon									
Weight			√			√		√	
Door									
Plate	√			√					
Comb									
Pizza				√					
Pick& Place									

### Coefficient of determination analysis result

**Fig 6:**
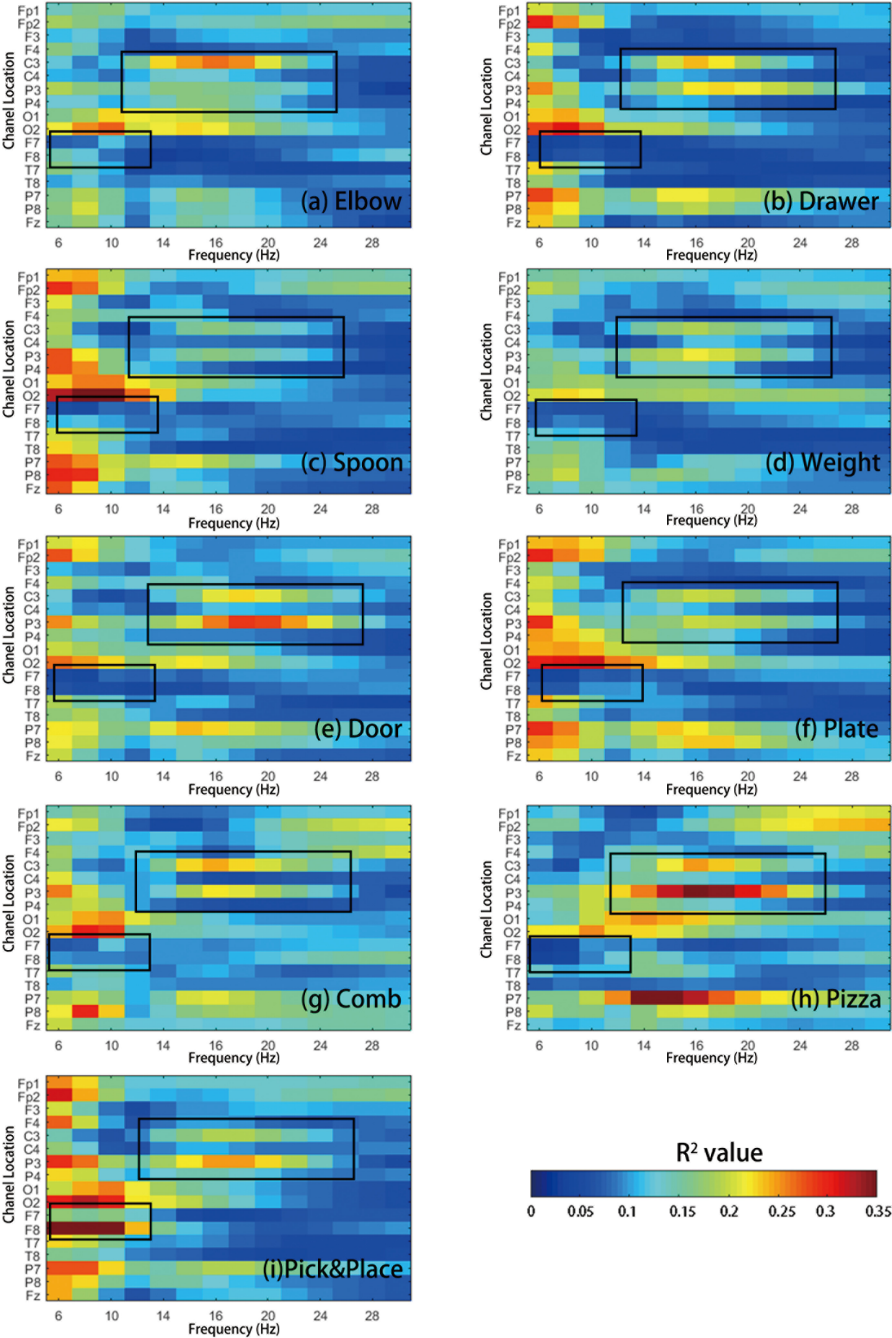
EEG R^2^ analysis for different motor imagery tasks, averaged among participants. (a) R^2^ value mapping for Rest Task vs Elbow Task; (b) R^2^ value mapping for Rest Task vs Drawer Task;(c) R^2^ value mapping for Rest Task vs Soup Task;(d) R^2^ value mapping for Rest Task vs Weight Task;(e) R^2^ value mapping for Rest Task vs Door Task(f) R^2^ value mapping for Rest Task vs Plate Task;(g) R^2^ value mapping for Rest Task vs Comb Task;(h) R^2^ value mapping for Rest Task vs Pizza Task;(i) R^2^ value mapping for Rest Task vs Pick&Place Task. Motor imagery related activities with high R2 value was labeled with a black box.

The averaged R^2^ value for different tasks is shown in Fig 6. One of our participants (H5) was left handed. The channels of his EEG were therefore flipped between left and right hemisphere in this analysis.

**Fig 7:**
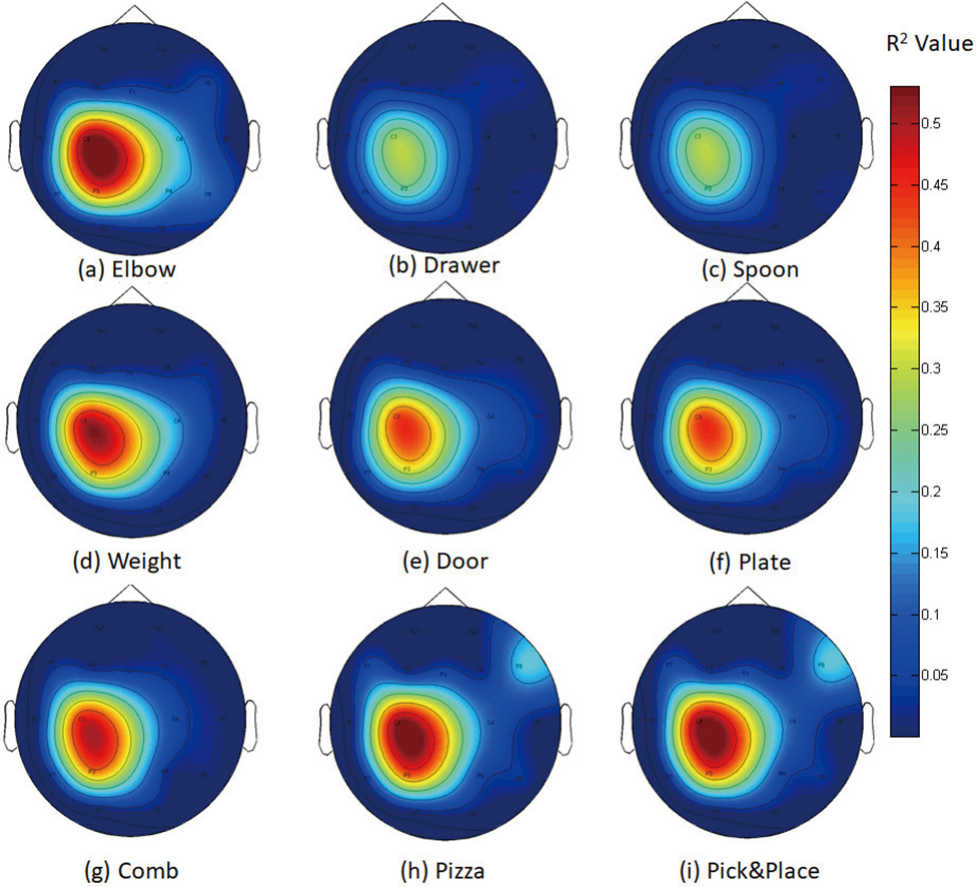
Topographical distribution of R^2^ value for H10 at 16Hz. (1) R^2^ value for Rest vs Elbow Task;(2) R^2^ value for Rest vs Drawer Task; (3) R^2^ value for Rest vs Soup Task; (4) R^2^ value for Rest vs Weight Task; (5) R^2^ value for Rest vs Door Task; (6) R^2^ value for Rest vs Plate Task; (7) R^2^ value for Rest vs Comb Task; (8) R^2^ value for Rest vs Pizza Task; (9) R^2^ value for Rest vs Pick & Place Task.

From Fig 6, we can see that most of the EEG activities are located in central and parietal lobe area. Most of the EEG activities for different motor imagery tasks (at C3 channel) are located around 12-20Hz. The peak activities for all the motor imagery tasks were always centered around 18Hz in C3 and P3 channel. Also, some activities were found in the F8 channel between 6-16Hz, which might be related to the motor planning [64,65]. Since all these two activities were both been seen around 16Hz, the topography analysis of 16Hz is shown in Fig 7, with H10, who had the highest cross-validation accuracy during the training among our participants.

In Fig 7, large R^2^ values are observed at electrode locations near the contralateral motor cortex area in all the motor imagery tasks. This was a result of the event-related desynchronization of the beta rhythms when motor imagery tasks were executed. The strength of activation and the topographical distribution, however, were different from task to task.

For H10, the topographical distributions for Rest vs Elbow Task and Rest vs Spoon Task are similar (see Fig 7(2) and 7(3)). Similar topographical distribution was observed in Door Task and Plate Task (Fig 7(5) and 7(6)), as well as Pizza Task and Pick&Place Task (Fig 7(8) and 7(9)). Especially, in Fig 7(8) and 7(9), while imagining to perform the Pizza Task and Pick&Place Task, EEG activity was recorded in the frontal lobe area (F8 channel), which might be related to the motor planning activities in complex motor imaginary tasks. These similarities suggested fundamental brain activity connections in performing some imagination tasks.

### Assessing the validity of the BP/CSP/FBCSP+LDA/DAL method during intra-task testing

**Fig 8:**
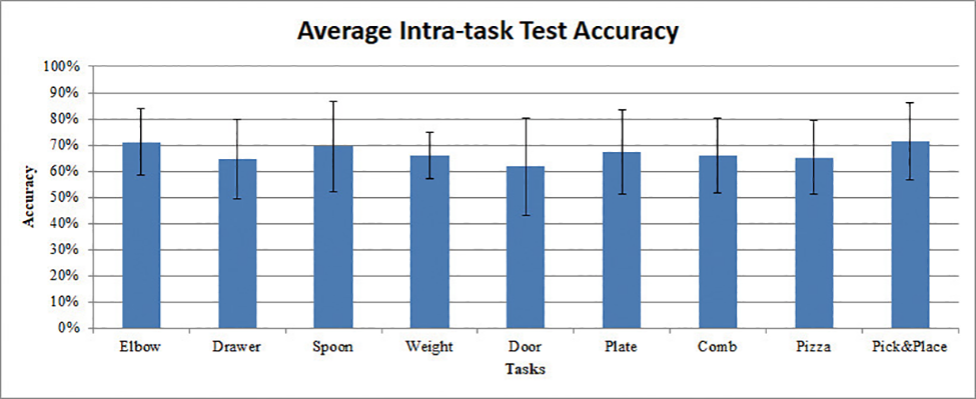
Average intra-task test accuracies for different motor imagery tasks.

For the intra-task problem, the models were generated and tested as described in Table 6. Although we performed a 5-fold cross validation in the training, we only reported the testing accuracy to keep the manuscript concise. The classification accuracy for each motor imagery task was averaged across participants (see Fig 8).

As shown in Fig 8, the Pick&Place task had the highest average intra-task test accuracy (0.715±0.148) among all the motor imagery tasks, followed by Elbow task (0.711±0.128). However, the difference between different tasks is not statistically significant (one-way ANOVA, p = 0.817). The door task, on the other hand, had the lowest average intra-task test accuracy (0.618±0.186). The average intra-tasks testing result shows the test accuracy was significantly higher than random (accuracy higher than 0.6359, p = 0.05 according to Muller-putz et al. [66]), except for the door task. All tasks showed higher accuracy than chance level (accuracy higher than 0.6141, p = 0.1).

## Discussions

In Fig 2, all the nine motor imagery tasks focused on upper extremity activities, centered around elbow joint movement. These tasks can arguably be divided into three main categories: i) simple joint tasks (SJM, i.e. Fig 2(B), Fig 2(C) and Fig 2(E)); ii) simple elbow joint that are commonly executed in everyday life and require a relatively low level of synergy of other joints (DSJM, i.e. Fig 2(F), Fig 2(G) and Fig 2(H)); and iii) and goal-oriented tasks (GOM, i.e. Fig 2(D), Fig 2(I) and Fig 2(J)), which require trajectory planning and multi-joint synergy.

The EEG performance varied across participants and the type of motor imagery task. GOM tasks such as Pick&Place Task and Pizza Task had a significantly higher accuracy compared to the SJM tasks. However, not all GOM tasks investigated in this study had higher cross-validation accuracy (e.g., Soup Task). In the Pizza Task and the Pick&Place Task, some activities were found from the F8 channel in lower frequency, which might be related to the motor planning activity [50][51]. More precise neural recordings would be needed to verify the brain region involved in order to confirm the activities in these tasks. However, it is surprising to see the Soup Task did not inducing similar activities in the same frequency band (in Fig 6(C)). This phenomenon may be due to the task design. We can see from Fig 6(C) that the highest R^2^ value is located in the O2 area, which suggests the Soup Task may be primarily related to vision/target related activity[67].

In the R^2^ analysis, the peak R^2^ value for the SJM tasks is generally smaller, and the contrast of the R^2^ mapping is lower than DSJM and GOM tasks. The “low-contrast” feature may result in the lower accuracy in cross-validation and intra-task test for models generated from the SJM tasks. While the difference is not statistically significant, this “low-contrast” feature might be a general pattern for upper extremity motor imagery. This could explain why the SJM tasks have higher inter-task test accuracy among all the other tasks (i.e. the EEG model generated from the SJM tasks are more versatile). For the SJM tasks, only the elbow joint was involved. All the three SJM tasks were similar. The only difference was the resistance feedback in these tasks. For example, in the Weight Task, because of the imagination of the weight, the Weight Task showed higher P3 activities than C3 activities. That might explain why the EEG model from the Weight Tasks exhibited higher versatility than DSJM and GOM tasks. For the Weight Task, there was only a 6% mean accuracy decrease between testing with data from its own task and the other tasks.

It is interesting to see how imagined interaction with other objects induces parietal lobe activities[68], such as the R^2^ value mapping varies in Elbow Task and Weight Task. The movement is physically almost the same, however, by just imaging a dumbbell in the hand excites brain activities around the P3 area.

It is also important to investigate the possibility of multi-class classification using the tasks mentioned in this paper in the future.

## Conclusion

In this study, we found that EEG models generated from single joint movements motor imagery tasks show higher versatility than other tasks. Among all the tested tasks, the Weight Task showed a statistically higher versatility than the other tasks (p<0.05) with the average inter-task testing accuracy was 0.605±0.022. Also, the other two single joint motor imagery tasks (i.e. Elbow Task and Drawer Task) showed higher versatility compared to non-single joint tasks. However, the difference was not statistically significant (p>0.05). The inter-task testing accuracy for the Elbow Task and Drawer Tasks was 0.594±0.022 and 0.590±0.022, respectively. Among the single joint motor imagery tasks, the difference was not statistically significant (ANOVA, p>0.05). For applications like rehabilitation, it would be possible for the individuals to go through an EEG training session that only involves the motor imagery of simple one-joint movements. The EEG model generated could then be re-used to classify different other goal-oriented motor imagery tasks.
